# Comparison of IORT (Radical and Boost Dose) and EBRT in Terms of Disease-Free Survival and Overall Survival according to Demographic, Pathologic, and Biological Factors in Patients with Breast Cancer

**DOI:** 10.1155/2021/2476527

**Published:** 2021-04-16

**Authors:** Solmaz Hashemi, Seyedmohammadreza Javadi, Mohammad Esmaeil Akbari, Hamidreza Mirzaei, Seied Rabi Mahdavi

**Affiliations:** ^1^Tabriz University of Medical Sciences, General Surgery Department, Tabriz, Iran; ^2^Cancer Research Center Shahid Beheshti, University of Medical Sciences, Tehran, Iran; ^3^Hamadan University of Medical Sciences, General Surgery Department, Hamadan, Iran; ^4^Shahid Beheshti University of Medical Sciences, Cancer Research Center, Tehran, Iran; ^5^Department of Medical Physics, Faculty of Medicine, Iran University of Medical Sciences, Tehran, Iran

## Abstract

**Background:**

The standard treatment for breast cancer is breast-conserving surgery (BCS) with radiotherapy. If external beam radiation therapy (EBRT) can be safely replaced with intraoperative radiotherapy (IORT), it will help patients to save their breast and to have equivocal or better results in DFS and overall survival (OS).

**Methods:**

A total of 2022 patients with breast cancer treated during 6 years were enrolled in the current study. A total of 657, 376, and 989 patients received EBRT, radical, and boost dose by IORT, respectively, according to the IRIORT consensus protocol. The primary endpoint was recurrence and death. The secondary endpoint was the role of variables in recurrence and death.

**Results:**

With a mean follow-up of 34.5 and 40.18 months for the IORT and EBRT groups, respectively, there was a significant difference in DFS between electron boost and X-ray boost groups (*P*=0.037) and the electron radical group compared with EBRT (*P*=0.025), but there was no significant difference between other boost and radical groups in DFS and OS.

**Conclusions:**

IORT can be a preferred treatment modality because of its noninferior outcomes, and in some special conditions, it has superior outcomes compared to EBRT, particularly in delivering radical dose with IORT.

## 1. Background

In the 19^th^ century, breast cancer was treated by invasive surgical approach, which was described by Halstead as a radical mastectomy [1]. With the introduction of breast-conserving surgery (BCS), this method, along with radiotherapy, came to be considered as the standard in breast cancer surgery. During this time, various studies with more than 20 years of follow-up showed no difference in treatment outcomes between BCS with radiotherapy and modified radical mastectomy [2–4]. Even in recent studies, it has been shown that preserving the breast can save the lives of patients with breast cancer [5]. Radiotherapy in BCS is of high importance for obtaining the ideal results in terms of recurrence and survival [6–8]. Radiotherapy can eradicate residual tumor cells by single- or double-stranded breakage of DNA and creation of free radicals [9]. Patients receiving external whole-breast radiotherapy (WBRT) received 45–50 Gy of radiation in 25 fractions over 5-6 weeks, followed by a booster of 10 Gy in 5 fractions. Because of the length of radiotherapy, some patients eschew BCS and prefer mastectomy [10]. Furthermore, radiation can damage adjacent organs such as the lungs and heart, and acute and chronic complications such as erythema, burns, skin dryness, fibrosis, fat necrosis, and telangiectasia can occur [11].

Intraoperative radiotherapy (IORT) is an accelerated partial breast irradiation (APBI) technique delivered at the time of lumpectomy. It has manifold advantages such as giving radiation therapy to the well-vascularized and oxygenated bed of tumor, the limitation of tumor cell repopulations, reduction in cytokine production, giving a minimum dose of radiation to adjacent organs, and patient convenience [12]. In 2009, the American Society of Radiation Oncology (ASTRO) consensus published a guideline for APBI and recommended three categories (suitable, cautionary, and unsuitable) for patient selection.

In a randomized noninferiority trial, Vaidya et al. compared IORT (20–50 kV) with WBI (40–56 Gy ± boost). Two-thirds of patients received IORT at the time of lumpectomy (prepathology group) and one-third received IORT after the preparation of the pathology report (postpathology group). At 29 months of follow-up, TARGIT-A had a higher rate of local recurrence in the IORT group (3.3% vs. 1.3%; *P*=0.04). Local recurrence was significantly different in the postpathology group (5.4% vs. 1.7%; *P*=0.07), but not in the prepathology group (2.1% vs. 1.1% *P*=0.3), and there was no difference in overall survival (OS) [13].

Veronesi et al. compared IORT (21 Gy-6–9 MeV) with WBI (50 Gy/25 fractions + 10 Gy boost). A 21 Gy with 6–9 MeV electron was delivered to 1305 women aged 48–75 years old with a tumor size of ≤2.5 cm. With a mean follow-up of 5.8 years, local recurrence had a higher rate (4.4% vs. 0.4%; *P* < 0.0001), and there was no difference in survival (96.8% vs. 96.9%) [14].

In this study, we delivered IORT with both X-ray and electrons and divided each group into radical and boost dose subgroups according to the IRIORT (Islamic Republic intraoperative radiotherapy) consensus. Then, we compared the efficacy of this modality with the control group that received WBRT.

## 2. Materials and Methods

A total of 2022 patients with breast cancer treated with BCS in three centers under the supervision of the Cancer Research Center of the Shahid Beheshti University of Medical Sciences (Tehran, Iran) were enrolled between September 2013 and September 2019. In each center, the technique of radiotherapy was different. The eligibility of patients to receive the radical or boost dose of IORT was determined by patients' demographic, pathologic, and biological factors according to the IRIORT consensus (Table 1).

The patients were divided into the following groups:

  The first group: BCS was performed for 657 patients. They received 45–50 Gy external beam radiation therapy (EBRT) in 25 fractions for 5-6 weeks and then 10 Gy boost dose in 5 fractions.
  The second group: intraoperative electron radiation therapy (IOERT) was delivered to 1075 patients after the removal of the tumor. IOERT was performed, using LIAC (light intraoperative accelerator), a mobile linear accelerated delivering energy levels of the electron (6–12 MeV). The patients categorized as suitable and possible groups in the IRIORT consensus received 21 Gy as a radical dose; otherwise, they would receive 12 Gy as a boost dose by making flaps in breast tissue around the tumor cavity with a maximum thickness of 2 cm. The protection of the chest wall was achieved using lead discs. Supplemental EBRT was delivered for patients who received the boost dose. The second group was divided in this way into 2 subgroups of electron radical (21 Gy) and electron boost (12 Gy).
  The third group: after doing BCS for 375 patients and the assurance of margin status in the frozen section, 20 Gy intraoperative X-ray radiation therapy (IOXRT) of 50 kV was delivered to breast tissue around the tumor cavity. IOXRT was performed using INTRABEAM ZEISS. If demographic, pathologic, and biological characteristics of the tumor, according to IRIORT (Islamic Republic intraoperative radiotherapy), were in suitable and possible groups, IORT would be mentioned as a radical dose and the patients would continue therapy by chemotherapy and hormone therapy. Otherwise, a boost dose would be used and the patient would need supplemental EBRT. The third group was divided into subgroups of X-ray radical and X-ray boost.

Patients with invasive breast cancer would be excluded from the study if they had metastatic disease, refused treatment, or did not continue the treatment. Patients were visited by the surgeon and radiooncologist every six months for up to two years. Next, mammography was performed yearly one year after the surgery and then every two years. If the interval visit time of every patient exceeded more than one year, telephone contact would be initiated to determine the last situation of the patients.

Disease-free survival (DFS) was defined as the time from diagnosis to the occurrence of recurrence (as local recurrence or distant metastasis in two groups of bone metastasis and other organ metastasis). OS was defined as the time from diagnosis to the last follow-up of the patient or the time of death.

Overall, 2022 patients were enrolled in this study, 657 of whom were assigned to the control group and received EBRT. A total of 1021 patients received IOERT, 706 of whom received the boost dose and 315 who received the radical dose. Of 344 patients who received IOXRT, 283 received the boost dose and 61 received the radical dose. Patients in the X-ray radical and electron radical groups were compared with stages 1 and 2 of the control group, and patients in the X-ray boost and electron boost groups were compared with stages 1, 2, and 3 of the control group.

The present longitudinal nonrandomized cohort study compared the recurrence and survival of the electron radical and X-ray radical groups with the EBRT group and the electron boost and X-ray boost groups with the EBRT group.

The primary endpoints were recurrence (local and distant) and death. The secondary endpoints were the role of age, tumor size, positive lymph nodes, grade, LVI, HR, Her2, and ki67 factors in recurrence and death.

Cumulative hazard function and survival plots were drawn using the Kaplan–Meier method. The log-rank test was used to evaluate the survival difference between the two treatment radiotherapy groups, as well. Hazard ratios (HRs) of the variables in DFS and OS were evaluated using a univariate Cox proportional hazards regression model. Only variables that were significant in levels 0 and 1 were evaluated with multivariate Cox proportional hazards regression. SPSS was used to analyze the data.

## 3. Results

As mentioned above, 989 patients received the boost dose of IORT, 706 and 283 of who were in the electron boost and X-ray boost groups, respectively.

Table 2 presents the characteristics of patients and tumors in the EBRT and IORT groups.

Local recurrence occurred in 2.7% (18 patients), 2.4% (17 patients), and 2.1% (6 patients) of the patients of the EBRT, electron boost, and X-ray boost groups, respectively. There was no significant difference among these groups. Bone metastasis constituted 2.3% (15 patients), 0.8% (6 patients), and 3.5% (10 patients) of the EBRT, electron boost, and X-ray boost groups, respectively. Bone metastasis occurred less in the group that received the electron boost. Other distant organ metastasis was 3.7% (24 patients), 2.8% (20 patients), and 3.5% (10 patients) in the EBRT, electron boost, and X-ray boost groups, respectively. Death occurred in 2.9% (19 patients), 2% (14 patients), and 2.1% (6 patients) in the EBRT, electron boost, and X-ray boost groups, respectively. Concerning death, there was no significant difference in these groups.

Table 3 presents factors associated with recurrence among patients that received a boost dose of IORT.

Patients receiving a boost dose of radiotherapy by IORT, stage, tumor size, grade, and hormone receptors had a significant difference in the increase of local recurrence risk (Table 3).

**Figure 1:**
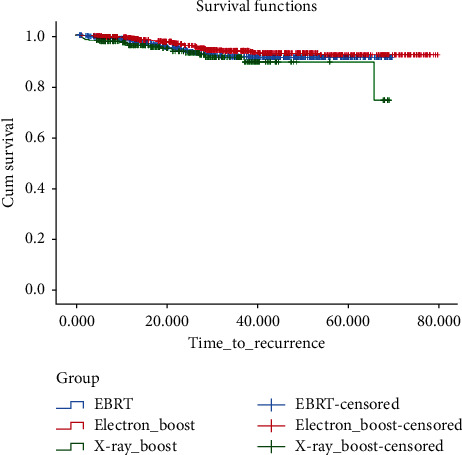
Cumulative incidence of disease-free survival in the boost groups in comparison with EBRT.

In the univariate analysis, there was a lower recurrence rate in stages 1 and 2 in comparison with stage 3 (HR: 0.32, 95% CI: 0.15–0.66, and HR: 0.42, 95% CI: 0.28–0.64; *P*=0.002), grade 2 tumors in comparison with grade 3 tumors (HR: 0.57, 95% CI: 0.38–0.86; *P*=0.008), hormone receptor positive tumors in comparison with hormone receptor negative tumors (HR: 0.61, 95% CI: 0.38–0.97; *P*=0.04), and tumor size of ≤2.5 cm in comparison with a tumor size of >3 cm (HR: 0.58, 95% CI: 0.36–0.92; *P*=0.021). In the multivariate analysis of these variables, grade 2 tumors had a lower recurrence rate with 44% HR in comparison with grade 3 tumors (*P*=0.038). The 5-year DFS for EBRT, electron boost, and X-ray boost groups was 91.3%, 92.3%, and 89.5%, respectively. There was no significant difference between electron boost and EBRT (*P*=0.26) and between X-ray boost and EBRT (*P*=0.36). However, there was a significant difference between electron boost and X-ray boost groups (*P*=0.037), and the electron boost group had a better DFS (Figure 1).

**Figure 2:**
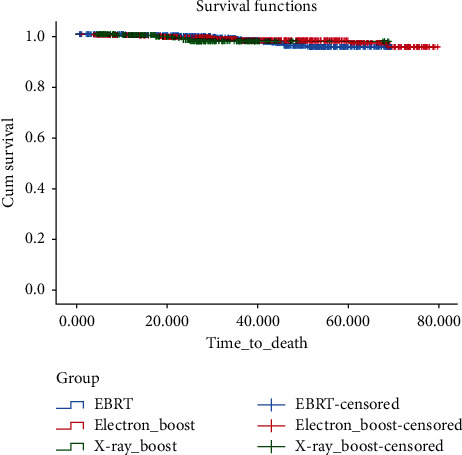
Cumulative incidence of overall survival in boost groups in comparison with EBRT.

The 5-year OS in EBRT, electron boost, and X-ray boost groups was 95.1%, 97.5%, and 97.2%, respectively. In terms of OS, there was no significant difference between the electron boost group and the EBRT (*P*=0.048), X-ray boost and EBRT (*P*=0.58) group, and the electron boost and X-ray boost group (*P*=0.58) (Figure 2).

In the univariate analysis, death showed a significant difference in stage 3 compared with stages 1 and 2 (HR: 0.32, 95% CI: 0.11–0.96, and HR: 0.23, 95% CI: 0.11–0.47; *P*=0.043), but had no significant difference in the multivariate analysis.

A total of 376 patients received a radical dose of IORT, 315 and 61 of who were in electron radical and X-ray radical groups, respectively.  Table 4 presents the characteristics of patients and tumors in the EBRT and IORT groups.

Local recurrence occurred in 1.9% (10 patients), 1% (3 patients), and 1.6% (1 patient) of the patients of the EBRT, electron radical, and X-ray radical groups, respectively. There was no significant difference in these groups. Bone metastasis was 1.9% (10 patients), 0%, and 1.6% (1 patient) in the EBRT, electron radical, and X-ray radical groups, respectively. Bone metastasis did not occur in the electron radical group.

Other distant organ metastasis was 2.3% (12 patients), 1% (3 patients), and 2.3% (2 patients) in the EBRT, electron radical, and X-ray radical groups, respectively. Also, there was a lower rate of other organ metastasis in the electron radical group. Death occurred in 1.4% (7 patients), 0.6% (2 patients), and 1.6% (1 patient) of the patients of the EBRT, electron radical, and X-ray radical groups, respectively. A lower death rate occurred in the electron radical group.

Table 5 presents the factors associated with recurrence among patients who received a radical dose of IORT.

In a univariate analysis of variables in patients receiving a radical dose of radiotherapy by IORT, tumor size (HR: 0.38, 95% CI: 0.16–0.90; *P*=0.029) and electron beam (HR: 0.37, 95% CI: 0.15–0.91; *P*=0.031) had a significant difference in the increase of local recurrence risk, but there was no significant difference in the multivariate analysis. The 5-year DFS of EBRT, electron radical, and X-ray radical groups was 93.5%, 96.7%, and 91.9%, respectively.

**Figure 3:**
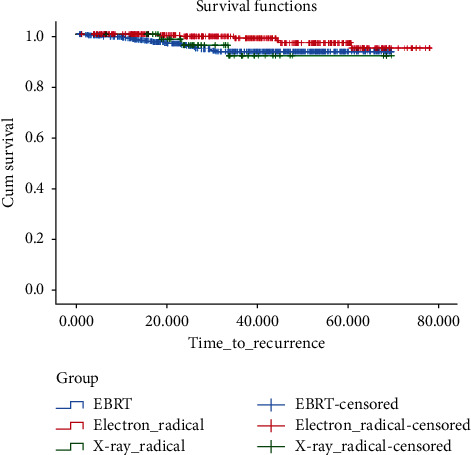
Cumulative incidence of disease-free survival in the radical groups in comparison with EBRT.

There was a significant difference in DFS between the electron radical group and EBRT (*P*=0.025), but there was no significant difference between the X-ray radical group and EBRT (*P*=0.92) and the electron radical and X-ray radical groups (*P*=0.07) (Figure 3).

**Figure 4:**
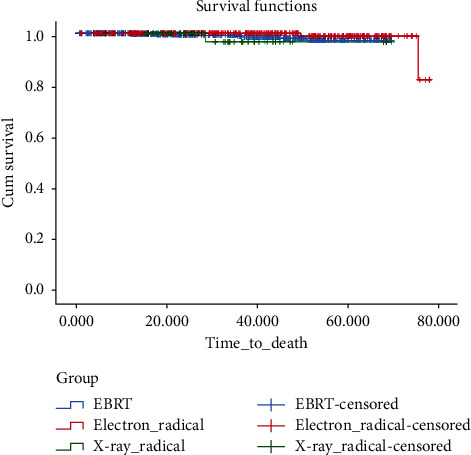
Cumulative incidence of overall survival in the radical groups in comparison with EBRT.

The 5-year OS in EBRT, electron radical, and X-ray radical groups was 97.3%, 98.9%, and 96.8%, respectively. In terms of OS, there was no significant difference between the electron radical group and EBRT (*P*=0.15), the X-ray radical group and EBRT (*P*=0.63), and the electron radical group and the X-ray radical group (*P*=0.051) (Figure 4).

In the univariate analysis of variables, there was a significant difference in OS in patients with hormone receptor positive in comparison with hormone receptor negative patients (HR: 0.25, 95% CI: 0.07–0.88; *P*=0.032). But, there was no significant difference in the multivariate analysis.

## 4. Discussion

IORT not only has a noninferior outcome in comparison with EBRT but also has a superior outcome under other conditions. In the present study, IORT, as a boost dose, does not show a significant difference with EBRT in terms of DFS and OS. However, stage, tumor size, grade, and hormone receptors of the tumor showed a significant difference in the increase of local recurrence risk.

In an analysis conducted in our center on locally advanced patients after neoadjuvant chemotherapy, in the IORT of patients with the photon in comparison with the electron as a boost dose and EBRT, the rate of events was lower, although there was no significant difference [15]. The biological effects of IORT consist of single- and double-stranded breakage of DNA, limitation of tumor cell repopulations, and reduction in cytokine production that affects the tissue microenvironment and immune system [16]. Relative biological effectiveness is a change in tissue microenvironment that is not favorable to invasion or tumor growth.

A single large dose of radiotherapy causes an immune response that regresses tumor growth in areas that are not irradiated or in distant metastasis. Mole called it the “abscopal effect” in 1953 as the antitumor effect of radiotherapy in a site other than the primary site of the tumor [17, 18]. Thus, IORT acts like a vaccine that stimulates the immune system and protects the patient against cancer.

We conducted a study in the cancer research center on 968 patients with breast cancer (IDC and ILC) to compare IOERT (a boost dose) with EBRT. The findings suggested that IOERT was noninferior in comparison with EBRT (*P*=0.215) [19].

Multiple studies evaluated IORT when it was used as the boost dose. One was Chang's study, which enrolled 55 patients to receive 5 Gy 50 kV X-rays. At a mean follow-up of 3.3 years, they had no local recurrence [20].

These findings of this study were in line with a study carried out by Fitedastner et al., who enrolled 1109 patients who received 10 Gy electrons as the boost dose. With six years of follow-up, the local recurrence rate was 0.8% [21].

Vaidya et al. enrolled 299 patients to receive 20 Gy 50 kV X-ray as boost dose like the TARGIT method. At 60.5 months of follow-up, the locoregional recurrence rate was 1.7% [22]. So, it seems that giving a boost dose of radiotherapy by IORT is an acceptable method.

In the present study, DFS in patients, who received IOERT as a radical dose, had a significant statistical difference with EBRT (*P*=0.025), but not in the IOXRT group and the univariate analysis of variables; tumor size had a significant difference in the increase of local recurrence risk (*P*=0.029). OS showed no significant difference in groups that received a radical dose of IORT in comparison with the EBRT group; however, hormone receptors of tumor caused a significant difference in the OS (*P*=0.032), which was not established by multivariate analysis.

TARGIT-A and ELIOT trials were based on 50 Gy low kV energy and high-voltage electron beam, respectively. They evaluated local recurrence in comparison with EBRT. Local recurrence rate for TARGIT-A and ELIOT was 3.3 vs. 1.3% (*P*=0.3) and 4.4% vs. 0.4% (*P* < 0.0001) [20], respectively [13, 14].

In a recent study, 1153 patients received delayed targeted IORT, and local recurrence and survival were compared with EBRT. Patients were younger than 45 years old with a tumor size of ≤3.5 cm. With a mean follow-up of 9 years, there was no statistically significant difference in local recurrence-free survival (*P*=0.57), mastectomy-free survival (*P*=0.38), distant DFS (*P*=0.98), and OS (*P*=0.3) [23].

In another study conducted in our center, we compared local recurrence in IOERT as a radical dose with EBRT, and there was no significant recurrence between groups (*P*=0.335) [24]. Montpelier delivered 21 Gy IOERT for 42 patients between 2004 and 2007. With 6 years of follow-up, local recurrence was 9.5% [25]. A different approach to radiotherapy by doing IORT may thus be indicated.

## 5. Conclusions

IORT can be a preferred treatment modality to WBRT because of its noninferior outcomes, and in some conditions, using better patient selection for delivering a radical dose by IORT, it has superior outcomes compared to EBRT. Patient convenience is one advantage of IORT, in which the patient does not spend a great deal of time in radiotherapy centers for a long time, and it protects the patient from EBRT complications.

We could expand our descriptions for patient selection in the IORT groups and deliver IORT even to younger patients, larger tumor sizes, and other histologies, such as ILC and DCIS. We could develop IORT usage for in-breast tumor recurrences and use BCS with IORT in these cases. The era of PBI has represented a paradigm shift in the treatment of early-stage breast cancer similar to that of the introduction of BCS as an alternative to mastectomy.

## Figures and Tables

**Table 1:** IRIORT consensus protocol for radical dose.

Patients' factors	Suitable	Possible	Contraindicated
Age	50≤	45–50	<45
Tumor size	≤3	3–3.5	≥3.5
Margins	Negative	Negative	Positive
Grade	1 and 2	Any	—
LVI	Negative	Any	Positive
ER status	Positive	Any	—
Multicentricity	No	No	Yes
Multifocality	No	Yes	—
IDC	Yes	Yes	—
ILC	Yes	Yes	—
Pure DCIS	≤3 cm	3-4 cm	>4 cm
EIC	<25%	≥25%	Diffuse
Her2	Any	—	—
Ki67	<30%	≥30%	—
Nodal status	Negative	Negative (*i*−, *i*+)	Positive
Axillary surgery	SLNB	SLNB, ALND	—
Neoadjuvant treatment	Not allowed	Not allowed	If used

LVI, lymphovascular invasion; EIC, extensive in situ component; SLNB, sentinel lymph node biopsy; ALND, axillary lymph node dissection; ILC, invasive lobular carcinoma; DCIS, ductal carcinoma in situ.

**Table 2:** Clinical, pathologic, and treatment-related characteristics of the EBRT and boost-IORT groups.

	EBRT			Electron boost		X-ray -boost		Total	
	*N*		%	*N*	%	*N*	%	*N*	%
	657		100	706	100	283	100	1646	100
Stage									
1	89		13.50	132	19	30	10.60	251	24.70
2	428		65.10	430	61.80	168	59.40	1026	57.40
3	140		21.30	134	19.30	85	30	359	17.90
Total	657		100	696	100	283	100	1636	100

Size									
≤2.5 cm	312		53.10	374	58.30	115	42.40	801	60.00
2.5 = 3 cm	144		24.50	99	15.40	39	14.40	282	17.00
>3 cm	132		22.40	169	26.30	117	43.20	418	23.10
Total	588		100	642	100	271	100	1501	100

Grade									
1	54		8.60	47	7.00	31	12.30	132	10.30
2	336		53.40	379	56.80	97	38.30	812	53.50
3	239		38	241	36.10	125	49.40	605	36.20
Total	629		100	667	100	253	100	1549	100

LVI									
Positive	254		42	328	49.90	118	72.40	700	43.00
Negative	351		58	329	50.10	45	27.60	725	57.00
Total	605		100	657	100	163	100	1425	100

Ki67									
>30%	32		11.80	176	29.00	23	35.90	231	27.10
≤30%	239		88.20	431	71.00	41	64.10	711	72.90
Total	271		100	607	100	64	100	942	100

ER									
Positive	372		76.40	517	72.30	175	72.30	1064	77.30
Negative	115		23.60	158	27.70	67	27.70	340	22.70
Total	487		100	675	100	242	100	1404	100

HER2									
Positive	91		23.80	89	14.00	68	25.50	248	19.10
Negative	292		76.20	547	86.00	199	74.50	1038	80.90
Total	383		100	636	100	267	100	1286	100

Age									
<40	160		24.90	135	20.90	88	31.10	383	19.80
=40–50	193		30.00	236	36.50	100	35.30	529	31.90
≥50	290		45.10	275	42.60	95	33.60	660	48.30
Total	643		100	646	100	283	100	1572	100

Histology									
IDC	591		90.90	449	64.50	161	62.60	1201	74.90
ILC	25		3.80	83	11.90	35	13.60	143	8.90
IDC + DCIS	23		3.50	93	13.40	39	15.20	155	9.70
IDC + ILC	2		0.30	60	8.60	19	7.40	81	5.10
Others	9		1.40	11	1.60	3	1.20	23	1.40

Adjuvant therapy									
CT + HT	245		68.40	435	72.00	166	70.00	846	70.60
HT alone	33		9.20	19	3.10	6	2.50	58	4.80
CT alone	80		22.30	150	24.80	65	27.40	295	24.60
Total	358		100	604	100	237	100	1199	100

**Table 3:** Factors associated with recurrence in patients that received boost dose by IORT (*P* value was considered concerning each variable that has the most risk).

		Electron boost		X-ray boost		*P* value
		*N*	%	*N*	%	
Stage	1	7/125	5.30	0/30	0.00	0.002
	2	18/412	4.20	9/159	5.40	—
	3	12/122	9.00	12/73	14.10	

Size	≤2.5 cm	14/360	3.70	6/109	5.20	0.021
	2.5 = 3 cm	6/93	6.10	1/38	2.60	0.96
	>3 cm	14/155	8.30	12/105	10.30	

Grade	1	2/45	4.30	2/29	6.50	0.12
	2	14/365	3.70	4/93	4.10	0.008
	3	19/222	7.90	13/112	10.40	

LVI	Positive	18/310	5.50	11/107	9.30	0.4
	Negative	18/311	5.50	3/42	6.70	

KI67	>30%	8/168	4.50	0/23	0.00	0.37
	≤30%	24/407	5.60	2/39	4.90	

HER2	Positive	5/84	5.60	5/63	7.40	0.85
	Negative	29/518	5.30	15/184	7.50	

ER	Positive	25/492	4.80	10/165	5.70	
	Negative	10/148	6.30	10/57	14.90	0.04

PR	Positive	19/431	4.20	0/0	0.00	
	Negative	9/159	5.40	0/0	0.00	0.19

Age	<40	7/128	5.20	6/82	6.80	0.09
	=40–50	14/222	5.90	10/90	10.00	0.56
	≥50	12/263	4.40	5/90	5.30	

**Table 4:** Clinical, pathologic, and treatment-related characteristics of the EBRT and radical-IORT groups.

	EBRT		Electron radical		X-ray radical		Total	
	*N*	%	*N*	%	*N*	%	*N*	%
	517	100	315	100	61	100	893	100
Stage								
1	89	17.20	222	70.90	24	40.00	335	37.60
2	428	82.80	91	29.10	36	60.00	555	62.40
Total	517	100	313	100	60	100	890	100

Size								
≤2.5 cm	237	52.70	276	88.20	46	78.00	559	68.00
2.5 = 3 cm	83	18.40	25	8.00	11	18.60	119	14.50
>3 cm	130	28.90	12	3.80	2	3.40	144	17.50
Total	450	100	313	100	59	100	822	100

Grade								
1	46	9.30	50	16.80	15	26.30	111	13.10
2	261	52.70	181	60.70	25	43.90	467	54.90
3	188	38.00	67	22.50	17	29.80	272	32.00
Total	495	100	298	100	57	100	850	100

LVI								
Positive	188	39.70	35	11.90	10	76.90	233	29.80
Negative	286	60.30	260	88.10	3	23.10	549	70.20
Total	474	100	295	100	13	100	782	100

Ki67								
>30%	27	12.20	101	36.50	0	0.00	128	25.40
≤30%	195	87.80	176	63.50	4	100.00	375	74.60
Total	222	100	277	100	4	100	503	100

ER								
Positive	372	76.40	256	83.90	39	81.30	667	79.40
Negative	115	23.60	49	16.10	9	18.80	173	20.60
Total	487	100	305	100	48	100	840	100

HER2								
Positive	91	23.80	52	18.30	11	19.30	154	21.30
Negative	292	76.20	232	81.70	46	80.70	570	78.70
Total	383	100	284	100	57	100	724	100

Age								
<40	110	21.90	3	1.00	0	0.00	113	12.90
=40–50	166	33.00	72	22.90	19	31.70	257	29.30
≥50	227	45.10	239	76.10	41	68.30	507	57.80
Total	503	100	314	100	60	100	877	100

Histology								
IDC	483	94.50	218	70.10	39	79.60	740	85.00
ILC	20	3.90	53	17.00	5	10.20	78	9.00
IDC + DCIS	0	0.00	19	6.10	0	0.00	19	2.20
IDC + ILC	0	0.00	17	5.50	5	10.20	22	2.50
Others	8	1.60	4	1.30	0	0.00	12	1.40

Adjuvant therapy								
CT + HT	245	68.40	117	51.10	26	65.00	388	61.90
HT alone	33	9.20	78	34.10	7	17.50	118	18.80
CT alone	80	22.30	34	14.80	7	17.50	121	19.30
Total	358	100.00	229	100.00	40	100.00	627	100.00

**Table 5:** Factors associated with recurrence in patients that received radical dose by IORT (*P* value was considered concerning each variable that has the most risk).

		Electron radical		X-ray radical		*P* value
		*N*	%	*N*	%	
Stage	1	4/218	1.80	2/22	8.30	0.08
	2	2/89	2.20	135	2.80	

Size	≤2.5 cm	5/271	1.80	2/44	4.30	0.02
	2.5 = 3 cm	1/24	4.00	1/10	9.10	

Grade	1	1/49	2.00	0/15	0.00	0.15
	2	3/178	1.70	2/23	8.00	0.71
	3	2/65	3.00	1/16	5.90	

LVI	Positive	1/34	2.90	0/10	0.00	0.58
	Negative	5/255	1.90	0/3	0.00	

Ki67	>30%	4/97	4.00	0/0	0.00	0.95
	≤30%	2/174	1.10	0/4	0.00	

HER2	Positive	0/52	0.00	0/11	0.00	0.4
	Negative	6/226	2.60	3/43	6.50	

ER	Positive	5/251	2.00	0/39	0.00	0.25
	Negative	1/48	2.00	1/8	11.10	

PR	Positive	5/223	2.20	0/0	0.00	0.14
	Negative	1/58	1.70	0/0	0.00	

## Data Availability

The data used to support the findings of this study are available from the corresponding author upon request.
